# mTORC1 is involved in the regulation of branched‐chain amino acid catabolism in mouse heart

**DOI:** 10.1002/2211-5463.12007

**Published:** 2016-01-04

**Authors:** Hongmin Zhen, Yasuyuki Kitaura, Yoshihiro Kadota, Takuya Ishikawa, Yusuke Kondo, Minjun Xu, Yukako Morishita, Miki Ota, Tomokazu Ito, Yoshiharu Shimomura

**Affiliations:** ^1^ Laboratory of Nutritional BiochemistryDepartment of Applied Molecular BiosciencesGraduate School of Bioagricultural SciencesNagoya UniversityNagoya, Japan; ^2^ Laboratory of BiomacromoleculesDepartment of Applied Molecular BiosciencesGraduate School of Bioagricultural SciencesNagoya UniversityNagoya, Japan

**Keywords:** branched‐chain amino acid, branched‐chain α‐ketoacid dehydrogenase complex, leucine, mTORC1

## Abstract

The branched‐chain α‐ketoacid dehydrogenase (BCKDH) complex regulates branched‐chain amino acid (BCAA) catabolism by controlling the second step of this catabolic pathway. In the present study, we examined the *in vivo* effects of treatment with an mTORC1 inhibitor, rapamycin, on cardiac BCKDH complex activity in mice. Oral administration of leucine in control mice significantly activated the cardiac BCKDH complex with an increase in cardiac concentrations of leucine and α‐ketoisocaproate. However, rapamycin treatment significantly suppressed the leucine‐induced activation of the complex despite similar increases in cardiac leucine and α‐ketoisocaproate levels. Rapamycin treatment fully inhibited mTORC1 activity, measured by the phosphorylation state of ribosomal protein S6 kinase 1. These results suggest that mTORC1 is involved in the regulation of cardiac BCAA catabolism.

**Abbreviations**
BCAA: branched‐chain amino acid
BCAT: branched‐chain aminotransferase
BCKA: branched‐chain α‐ketoacid
BCKDH: branched‐chain α‐ketoacid dehydrogenase
BDK: BCKDH kinase
BDP: BCKDH phosphatase
BW: body weight
CoA: coenzyme A
DMB: 1,2‐diamino‐4,5‐ methylene‐dioxybenzene
ECL: enhanced chemiluminescence
HPLC: high‐performance liquid chromatography
KIC: α‐ketoisocaproate
mTORC1: mammalian target of rapamycin complex 1
PAGE: polyacrylamide gel electrophoresis
PEG: polyethylene glycol
PVDF: polyvinylidene difluoride
S6K1: ribosomal protein S6 kinase 1
TPCK: phenylalanyl chloromethyl ketone

Branched‐chain amino acids (BCAAs), including leucine, isoleucine and valine, are essential amino acids in mammals and must be continuously acquired from the diet. As BCAAs regulate protein synthesis 1, 2, glucose metabolism 3, and neurotransmitter synthesis 4, the catabolism of BCAAs must be tightly regulated. The first two steps of the catabolic pathway are common to the three BCAAs. Branched‐chain aminotransferase (BCAT) catalyses the first reversible transamination of BCAAs to produce branched‐chain α‐ketoacids (BCKAs), and the branched‐chain α‐ketoacid dehydrogenase (BCKDH) complex catalyses the second irreversible oxidative decarboxylation of BCKAs to form corresponding coenzyme A (CoA) esters 5. The BCKDH complex catalyses the rate‐limiting step of BCAA catabolism and therefore is likely to be tightly controlled.

The BCKDH complex is regulated by a phosphorylation‐dephosphorylation cycle. BCKDH kinase (BDK) is responsible for inactivation of the complex by phosphorylation of the E1α subunit of the complex 6, 7, and BCKDH phosphatase (BDP) is responsible for reactivation of the complex by dephosphorylation 8. BDK is considered to be a primary regulator of the BCKDH complex, because BDK activity is negatively correlated with the activity state of BCKDH complex 9. It was reported that protein starvation 10, diabetes 11, 12 and hormones 7, 13 influence BDK expression to regulate the BCKDH activity in the long term. In acute regulatory mechanisms, BDK is downregulated by the transamination products of BCAA (especially α‐ketoisocaproate (KIC)) in an allosteric manner, which is accompanied by dissociation of BDK from the complex 5, 9, 14.

Among BCAAs, leucine is the most physiologically active amino acid, because it is not only an essential amino acid but also signals to mammalian target of rapamycin complex 1 (mTORC1), which in turn activates 70 kDa ribosomal protein S6 kinase (S6K1) to promote mRNA translation 15. However, the exact cellular mechanisms regulating leucine activation of the mTORC1 signalling system remain undefined. Some reports have proposed that leucine‐induced mTORC1 activation might be connected with the structural characteristics of leucine molecules 16 or the second regulatory step of leucine catabolism 17. However, Beugnet *et al*. 18 suggested that intracellular amino acid levels might be involved in mTORC1 activation, a finding supported by a study using cultured cells with enzymatic defects in leucine catabolism that showed that leucine‐induced activation of mTORC1 was related to leucine pool size and repletion capacity, but not leucine oxidation capacity 19. From these findings, the correlation between mTORC1 signalling and the leucine catabolic pathway does not seem to be physiologically important. However, it has been clearly demonstrated that an increase in the cellular concentration of leucine activates both mTORC1 signalling and the BCAA catabolic pathway. Therefore, further studies are required to clarify the relationship between these systems. In the present study, we used rapamycin‐treated mice to investigate the involvement of mTORC1 in the regulation of BCKDH complex activity.

## Materials and methods

### Materials

1,2‐Diamino‐4,5‐methylenedioxybenzene (DMB) was obtained from Dojindo Laboratories (Kumamoto, Japan). Lambda protein phosphatase was acquired from New England BioLabs (Beverly, MA, USA). Rabbit polyclonal antibodies against total‐S6K1, phospho‐S6K1 (Thr389) and goat anti‐rabbit IgG conjugated to horseradish peroxidase were purchased from Cell Signaling Technology (Danvers, MA, USA) or Bio‐Rad Laboratories (Hercules, CA, USA) for use in Western blot analyses. Antibody against BCKDH complex and BDK were prepared as described previously 12. Enhanced chemiluminescence (ECL) reagents and immobilon polyvinylidene difluoride (PVDF) membranes were purchased from Amersham Pharmacia Biotech (Little Chalfont, Buckinghamshire, UK). All other reagents were of analytical grade and were purchased from Wako (Osaka, Japan) or Sigma Aldrich Japan (Tokyo, Japan).

### Animals and experimental design

The Animal Care Committee of Nagoya University Graduate School of Bioagricultural Sciences approved all procedures for the animal experiments in this study. Male ddY mice (aged 7 weeks, ~ 34 g body weight) were obtained from Japan SLC Inc. (Hamamatsu, Japan) and were maintained in a conventional animal room with controlled temperature (22 ± 2 °C) and a 12 h light‐dark cycle (lighting on at 0800). They were fed a commercial diet (CE2; CLEA Japan, Tokyo, Japan) and tap water for 1 week *ad libitum* during acclimatization in the animal room and were then used in experiments.

Mice were fasted for 12 h before experiment initiation at 2300. Animals were arbitrarily divided into two groups: vehicle (Veh) and rapamycin (Rap) groups. Mice in each respective group were injected intraperitoneally with rapamycin 4.5 mg·kg^−1^ body weight (BW) or vehicle. The rapamycin was prepared by diluting a stock solution (11.25 mg·mL^−1^ in 99.5% (v/v) ethanol, stored at −30 °C) to a final concentration of 4% (v/v) ethanol in the vehicle immediately prior to use. The vehicle consisted of 5% polyethylene glycol (PEG)‐400 and 5% Tween 80 20.

Three hours after injection of vehicle or rapamycin, each group was subdivided into two subgroups: the saline (Veh/Sal or Rap/Sal) and leucine (Veh/Leu or Rap/Leu) subgroups. These subgroups of mice were orally administered saline (22.5 mL·kg^−1^ BW) or 2% leucine solution in saline (0.45 g leucine·kg^−1^ BW) 21, respectively. Thirty minutes after administration of saline or leucine, the mice were anesthetized with isoflurane and then sacrificed by collecting blood from the inferior vena cave to prepare plasma for measuring amino acid concentrations. The heart was then removed and immediately freeze‐clamped at liquid nitrogen temperature and stored at −80 °C until analyses.

### Amino acid analysis

Plasma samples were mixed with 3% sulfosalicylic acid to give a final concentration of 1.5% and then centrifuged at 700 ***g*** at 4 °C for 15 min to remove precipitated proteins. To prepare deproteinized tissue extracts, ~ 50 mg pulverized frozen heart was homogenized in 300 μL of 3% sulfosalicylic acid and centrifuged as described previously 22. The amino acid concentrations in the supernatants obtained were analysed using an automatic amino acid analyzer (JLC‐500/V; JEOL, Tokyo, Japan).

### Cardiac BCKA concentrations

Cardiac concentrations of BCKAs were analysed using HPLC 23. Briefly, ~ 30 mg pulverized frozen heart was homogenized in 180 μL of 0.8 m perchloric acid and then centrifuged at 18 000 ***g*** at 4 °C for 15 min to remove precipitated proteins. The BCKAs in the supernatant were reacted with DMB to produce fluorescent compounds that could be separated and quantified by the HPLC system (Shimadzu Co., Kyoto, Japan).

### Cardiac BCKDH complex activity

Cardiac BCKDH complex activity was measured as described previously 24. The enzyme activity was defined as the rate of formation of 1 μmol of NADH/min at 30 °C. Both actual and total activities were measured; the former corresponded to *in vivo* dephosphorylated enzyme levels and the latter was obtained by treating the tissue extract with lambda protein phosphatase to fully dephosphorylate enzymes 25. The activity state of the enzyme is defined as the percentage of actual activity relative to total activity 24.

### Abundance of the BDK bound to the BCKDH complex

The BDK bound to the BCKDH complex was quantified by the method of immunoprecipitation of BCKDH complex in the heart extract and Western blotting of BDK and the complex as described previously 25.

### Quantification of phospho‐S6K1 at Thr 389

Approximately 20 mg of pulverized frozen heart was homogenized in ice‐cold buffer (50 mm HEPES (pH 7.4 with NaOH), 1% (w/v) Triton X‐100, 1 mm EDTA, 1 mm EGTA, 2 mm Na_3_VO_4_, 100 mm NaF, 50 mm Na_4_P_2_O_7_, 1 mm phenylmethylsulfonyl fluoride, 20 μg·mL^−1^ leupeptin, 5 μg·mL^−1^ aprotinin, 0.1 mg·mL^−1^ trypsin inhibitor, 0.1 mm TPCK) using a motor‐driven Teflon homogenizer, and the homogenates were centrifuged at 8000 ***g*** at 4 °C for 15 min to remove debris. The extracts containing 20 μg protein were loaded for SDS/PAGE and transferred to PVDF membranes. The membranes were then probed with antibodies against S6K1 and phospho‐S6K1 (Thr 389) 21.

### Statistics

Values are expressed as means ± SEM. The data were analysed by a two‐way anova and the Tukey–Kramer method. *P* < 0.05 was considered significantly different.

## Results

### Plasma and cardiac BCAA concentrations

It was reported that plasma leucine concentrations showed a peak 30 min after oral administration of leucine in mice 26. Therefore, in the present study, we chose this time point to sacrifice the mice for collection of blood and heart. We confirmed that the plasma leucine concentration in Veh/Leu mice was markedly higher than that in Veh/Sal mice (197 ± 23 μm for Veh/Sal vs. 1479 μm for Veh/Leu). The same levels of plasma leucine were observed in Rap/Leu and Rap/Sal mice respectively.

Cardiac leucine concentrations in Veh/Leu and Rap/Leu mice were also significantly higher than those of Veh/Sal and Rap/Sal mice respectively (Table 1). The leucine concentrations were not significantly different between the Veh and Rap groups, but tended to be higher in the latter compared to the former (Table 1).

**Table 1:** Cardiac BCAA concentrations 30 min after leucine administration

BCAA	Veh (nmol·g^−1^ wet weight tissue)		Rap (nmol·g^−1^ wet weight tissue)	
	Sal	Leu	Sal	Leu
Leucine	123 ± 21^a^	563 ± 70^b^	155 ± 8^a^	485 ± 25^b^
Isoleucine	97 ± 9^a^	60 ± 4^b^	122 ± 2^c^	68 ± 6^b^
Valine	131 ± 12^a,c^	67 ± 13^b^	145 ± 8^a^	80 ± 13^b,c^

Values are means ± SE, *n* = 5. Means not sharing the same superscript letters in the same row are significantly different, *P* < 0.05.

Cardiac concentrations of isoleucine and valine in Veh/Leu mice and Rap/Leu mice were significantly lower than those in Veh/Sal mice and Rap/Sal mice respectively (Table 1). The concentrations of these amino acids tended to be higher in the Rap group than in the Veh group.

### Cardiac BCKA concentrations

As KIC is an important physiological factor to activate the BCKDH complex through inhibition of BDK 5, we measured cardiac BCKA concentrations. Alterations in concentrations of the three BCKAs showed the same trends as those of the BCAA concentrations, although the BCKA concentrations were < 1% of the BCAA concentrations (Table 2).

**Table 2:** Cardiac BCKA concentrations 30 min after leucine administration

BCKA	Veh (nmol·g^−1^ wet weight tissue)		Rap (nmol·g^−1^ wet weight tissue)	
	Sal	Leu	Sal	Leu
KIC	0.23 ± 0.04^a^	0.78 ± 0.07^b^	0.34 ± 0.04^a^	1.16 ± 0.17^b^
KIV	0.24 ± 0.05^a^	0.10 ± 0.02^b^	0.24 ± 0.03^a^	0.18 ± 0.02^a,b^
KMV	0.14 ± 0.03	0.06 ± 0.01	0.17 ± 0.03	0.10 ± 0.03

Values are means ± SE, *n* = 5–6. Means not sharing the same superscript letters in the same row are significantly different, *P* < 0.05.

### Cardiac BCKDH complex activities

**Figure 1:**
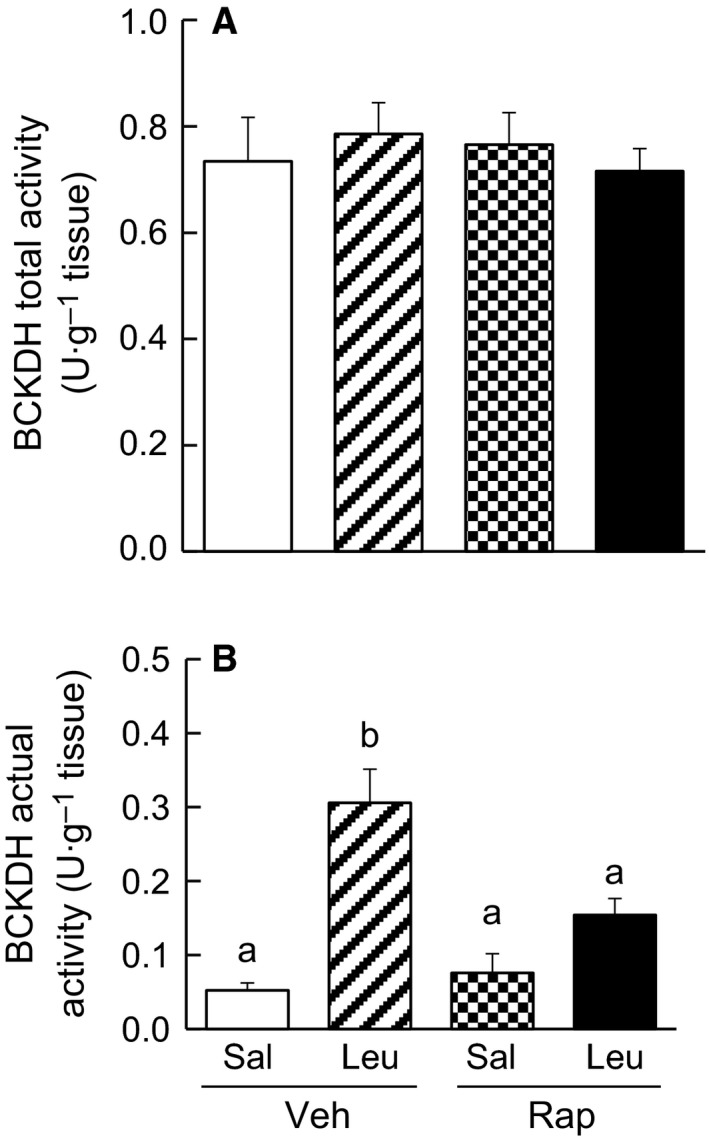
Cardiac BCKDH complex activity in mice. (A) Total and (B) actual activities of the cardiac BCKDH complex. Fasted mice were injected intraperitoneally with rapamycin (4.5 mg·kg^−1^ body weight) or vehicle. Three hours later, one‐half of each groups of mice were orally administrated saline or leucine. Hearts were collected from mice 30 min after oral administration of saline or leucine. Values are mean ± SE,* n* = 5–7. Means not sharing the same letters are significantly different, *P* < 0.05.

Cardiac BCKDH complex activity was measured in mice under the same conditions as described above. The total activities of the cardiac BCKDH complex were not different among the four groups of mice (Fig. 1A). The actual activity of the complex in Veh/Sal mice was as low as ~ 7% of the total activity, and that in Veh/Leu mice was significantly higher (~ 40% of the total activity) than that in Veh/Sal mice (Fig. 1B). The actual activity in Rap/Sal mice was the same as that in Veh/Sal mice, while activity in Rap/Leu mice tended to be higher, but was not significantly different from that in Rap/Sal, and thus was significantly lower than that in Veh/Leu mice (Fig. 1B).

### Abundance of the BDK bound to BCKDH complex

It has been reported that the BDK exists as two forms, a form bound to the BCKDH complex and a free form, and the former only is suggested to be active 25. Therefore, the abundance of bound BDK was measured in the immunoprecipitates with the antibody against the complex. The abundance of the bound BDK expressed by the ratio of BDK/E2 subunit (*n* = three in each group) was 1.00 ± 0.16 for Veh/Sal, 0.52 ± 0.02 for Veh/Leu, 0.67 ± 0.28 for Rap/Sal, and 0.67 ± 0.23 for Rap/Leu. Although there was no significant difference among four groups, the abundance of the bound BDK tended to be lower in Veh/Leu mice than in other groups of mice.

### Phosphorylation of S6K1

**Figure 2:**
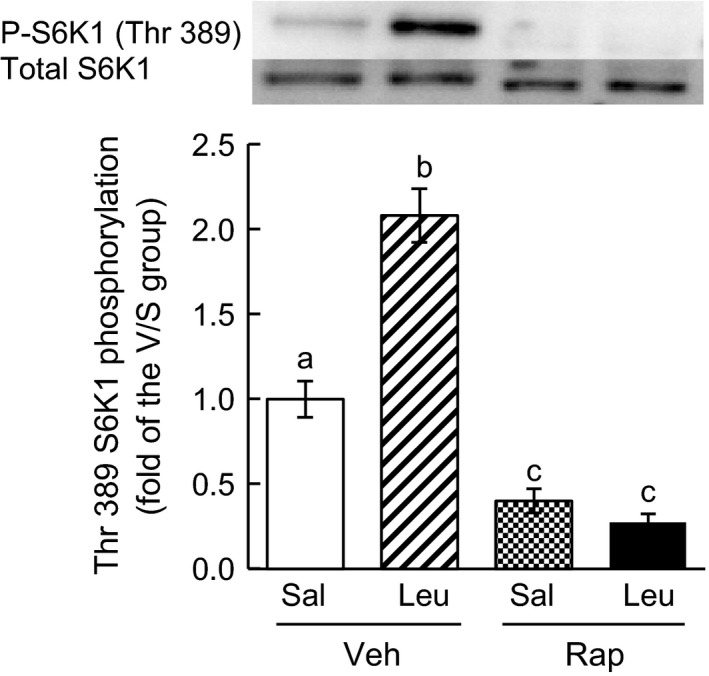
Phosphorylation of S6K1 Thr 389 in mouse heart. Total and phosphorylated S6K1 were measured by western blot analysis using anti‐total‐S6K1 and antiphospho‐S6K1 (Thr 389) antibodies, respectively. Representative data for total and phosphorylated S6K1 (P‐S6K1) are shown in the upper panel. The bar graph gives quantification of the relative amounts of phosphorylated S6K1 for each group (mean ± SE,* n* = 5–7). Means not sharing the same letters are significantly different, *P* < 0.05.

Phosphorylation of the Thr 389 site of S6K1 is catalysed by mTORC1; thus, phosphorylation was quantified to determine the activity of mTORC1. The phosphorylation state of S6K1 Thr 389 was significantly higher in Veh/Leu mice than in Veh/Sal mice (Fig. 2). On the other hand, the phosphorylation states in Rap/Sal and Rap/Leu mice were not different and were markedly lower than those in Veh/Sal and Veh/Leu mice (Fig. 2).

## Discussion

In this study, we showed that leucine administration significantly increased cardiac leucine concentrations and activated the cardiac BCKDH complex in control mice (Veh group). The leucine‐induced activation of the BCKDH complex was previously reported in rat skeletal muscle 27 and was explained through inhibition of the BDK by KIC formed from leucine 5. This explanation also accounts for the activation of the BCKDH complex observed in the present study, because the cardiac concentration of KIC was significantly increased following leucine administration. On the other hand, the leucine‐induced activation of the cardiac BCKDH complex in mice was significantly suppressed by treatment with rapamycin (Rap group), although cardiac concentrations of leucine and KIC were significantly increased by leucine administration. The rapamycin treatment almost completely abolished phosphorylation of S6K1 Thr 389, indicating that rapamycin fully inhibited cardiac mTORC1 *in vivo*. These findings suggest that leucine‐induced activation of the cardiac BCKDH complex may be, in part, mediated by mTORC1. This is the first report showing that mTORC1 may be involved in the regulation of BCAA catabolism because the BCKDH complex is the rate‐limiting enzyme in BCAA catabolism.

Lynch *et al*. 28 examined the responses of mTORC and BCKDH complex in rat adipose tissue to plasma leucine concentrations and found that mTORC activity, measured by phosphorylation of S6K1 Thr 389, was more sensitive to an increase in plasma leucine concentration than activation of the BCKDH complex, analysed by the phosphorylation state of BCKDH E1α Ser 293. It is concluded in this report that activation of the BCKDH complex is not required for activation of mTORC1 signalling. This conclusion is supported by the study of Schriever *et al*. using cultured cells 19. However, that study did not examine effects of mTORC1 activity on the activation of the BCKDH complex. In the present study, we demonstrated that mTORC1 may have a role in the activation of BCKDH complex and have therefore provided a new viewpoint for the relationship between mTORC1 signalling and BCAA catabolism. Furthermore, we observed that cardiac KIC concentrations tended to be higher in the rapamycin‐treated mice than in control mice, which is consistent with the suppression of the leucine‐induced activation of BCKDH complex by the rapamycin treatment. Even this high KIC concentration in rapamycin‐treated mice, the BCKDH complex was not significantly activated.

It is interesting to consider how mTORC1 mediates leucine‐induced activation of the BCKDH complex. The activity state of the complex *in vivo* should be determined by the balance of the activities between BDK and BDP. It has been reported that the bound BDK is an active form of the kinase and that its abundance is inversely correlated with the activity state of the BCKDH complex 9. In the present study, there was a tendency that the abundance of bound BDK was decreased by leucine administration and that this phenomenon was suppressed by the rapamycin treatment, suggesting that mTORC1 may act on BDK for activation of the complex. However, BDP is not ruled out of the mechanisms, because this phosphatase is an important enzyme for activation of the complex. Unfortunately, it is technically difficult to measure the BDP activity in tissue extracts.

It has been reported that leucine administration decreases plasma concentrations of isoleucine and valine in rats 21 and humans 29. In the present study, cardiac concentrations of isoleucine and valine were significantly decreased by leucine administration, suggesting that leucine regulates the three BCAA concentrations not only in the circulation but also in muscle tissues. The leucine‐induced activation of the cardiac BCKDH complex may contribute to the decreases in isoleucine and valine concentrations.

As reported previously, BCAAs, especially leucine, are physiologically active amino acids. Although the regulation of BCAA catabolism has been intensively studied 3, 9, the present study presented a novel viewpoint in the concept of the regulation of BCAA catabolism. Further studies are warranted to clarify the mechanisms responsible for the regulation of BCAA catabolism as well as the physiological and clinical functions of BCAAs.

## Author contributions

HZ, Y Kitaura, YS conceived and designed the experiments. The experiments were performed by HZ, Y Kitaura, Y Kadota, TI, Y Kondo, MX, TI. The data were analysed by HZ, Y Kitaura, TY, YS. The manuscript was written by YS, HZ, Y Kitaura.
